# A visualized dynamic prediction model for survival of patients with geriatric thyroid cancer: A population-based study

**DOI:** 10.3389/fendo.2022.1038041

**Published:** 2022-12-09

**Authors:** Ting-ting Zhang, Jing Zeng, Yan Yang, Jin-jing Wang, Yao-jie Kang, Dong-he Zhang, Xiao-zhu Liu, Kang Chen, Xuan Wang, Yi Fang

**Affiliations:** ^1^ Department of Endocrinology, The Fifth Medical Center of Chinese PLA General Hospital, Beijing, China; ^2^ Department of Day Clinic, The Fifth Medical Center of Chinese People's Liberation Army (PLA) General Hospital, Beijing, China; ^3^ Department of Cardiology, The Second Affiliated Hospital of Chongqing Medical University, Chongqing, China; ^4^ Department of Endocrinology, The First Medical Center of Chinese People's Liberation Army (PLA) General Hospital, Beijing, China

**Keywords:** thyroid cancer, geriatric patient, nomogram, prediction model, SEER

## Abstract

**Objective:**

Thyroid cancer (TC) is a common malignancy with a poor prognosis with aging. However, no accurate predictive survival model exists for patients with geriatric TC.We aimed to establish prediction models of prognosis in elderly TC.

**Methods:**

We retrospectively reviewed the clinicopathology characteristics of patients with geriatric TC in the Surveillance, Epidemiology, and End Results database (SEER) from 2004 to 2018. The risk predictors used to build the nomograms were derived from the Cox proportional risk regression. These nomograms were used to predict 1-, 3-, and 5-year overall survival and cancer-specific survival in elderly patients with TC. The accuracy and discriminability of the new model were evaluated by the consistency index (C-index) and calibration curve. The clinical applicability value of the model was assessed using the decision curve analysis.

**Results:**

We used the SEER database to include 16475 patients with geriatric TC diagnosed from 2004 to 2018. The patients from 2004 to 2015 were randomly sorted out on a scale of 7:3. They were classified into a training group (n = 8623) and a validation group (n = 3669). Patients with TC diagnosed in 2016–2018 were classified into external validation groups (n = 4183). The overall survival nomogram consisted of 10 variables (age, gender, marital status, histologic type, grade, TNM stage, surgery status, and tumor size). A cancer-specific survival nomogram consisted of eight factors (age, tumor size, grade, histologic type, surgery, and TNM stage). The C-index values for the training, validation, and external validation groups were 0.775 (95% confidence interval [CI] 0.785–0.765), 0.776 (95% CI 0.792–0.760), and 0.895(95% CI 0.873–0.917), respectively. The overall survival was consistent with a nomogram based on the calibration curve. Besides, the decision curve analysis showed excellent clinical application value of the nomogram. Additionally, we found that surgery could improve the prognosis of patients with geriatric at high-risk (P < 0.001) but not those at low-risk (P = 0.069).

**Conclusion:**

This was the first study to construct predictive survival nomograms for patients with geriatric TC. The well-established nomograms and the actual results could guide follow-up management strategies.

## Introduction

Undoubtedly one of the most common endocrine cancers is thyroid carcinoma (TC) (1). Despite its steady disease-specific mortality (0.5/100,000) (2), the TC incidence rate over the past 20 years has increased by approximately 2.5 times (5.57/100,000-13.98/100,000) (3). By 2030, TC is anticipated to be the fourth most prevalent cancer in the USA (4). However, the South Korean experience indicates that they will need to discourage early thyroid cancer discovery if they wish to stop their own “epidemic”.Vital statistics and cancer registry data for South Korea illustrate the effect of thyroid-cancer screening since 1999. Thyroid-cancer incidence increased rapidly after the turn of the century In 2011, the rate of thyroid-cancer diagnoses was 15 times that observed in 1993. This entire increase can be attributed to the detection of papillary thyroid cancer. Furthermore, despite the dramatic increase in incidence, mortality from thyroid cancer remains stable — a combination that is pathognomonic for over diagnosis (5).

With the continuously improved cancer prevention and treatment, the population aged ≥65 years will rise from 15% to 21% by 2030 in USA (6). In 2000, Americans aged ≥80 years represented approximately 3.3% of the population, which is expected to show a 2-fold increase by 2050 (7). Given the advanced age of patients with TC, we must consider the challenges that may arise as a direct result. In the 2015 Korean Central Cancer Registry, thyroid cancer was reported to be the fourth most common cancer in women aged 65 years or older (8).Recent literature shows that particularly poor prognosis is associated with age greater than 60 years (9, 10).Additionally, older individuals typically have more advanced-stage, aggressive, and widespread TC (11). Elderly individuals with TC often have follicular histology, vascular invasion, and extrathyroidal extension (12).In fact, Anaplastic thyroid cancer (ATC) is relatively more common with advanced age (13–16).. In several studies, older patients have had large volume tumor, lymph node metastasis, and distant metastasis at diagnosis and recurrence (17, 18). The survival rate was independently associated with a poorer prognosis starting at 60 years; elderly patients >70 years had the worst prognosis (12)..

TC has a satisfying good prognosis, with an average survival rate of 10 years for 90% of patients (19, 20). The median age of death for patients with TC is 73 years, and >70% of the deaths occur when patients are aged ≥65 years (21). A risk stratification technique that can enhance outcomes is required to predict overall survival (OS) for older patients with TC due to the limits of available treatment choices. Within this context, TNM staging can be used to determine the clinical staging of patients with cancer. However, the TNM classification is still insufficient in covering tumor biology and predicting all TC outcomes and treatment decisions made by elderly patients.

Nomograms have proved superior to the TNM staging system in different cancer studies (22, 23). A nomogram is a simple, user-friendly statistical prediction tool used to predict and quantify individual patient outcomes (24, 25). Population-based statistics show that, nevertheless, no study has created a nomogram of elderly TC persons. With the support of the Surveillance, Epidemiology, and End Results database (SEER) (26), we aimed to construct and verify a web-based survival prediction model for geriatric patients with TC. This model may be useful for individualized therapy, prognostic prediction, and follow-up strategy.

## Materials and methods

### Patient and screening criteria

The data of geriatric patients with TC were obtained using the SEER∗ Stat software (version 8.3.8). The timeframe for data collection was from 2004 to 2018.

The inclusion criteria were as follows: (1) patients aged ≥65 years; (2) positive histological diagnosis of TC by the 3rd Edition of the International Classification of Diseases for Oncology (ICD-O-3) without an autopsy or death certificate; (3) AJCC stage I–III with a histological grade I–III; (4) a positive follow-up.

The following exclusion criteria were used: (1) patients who had a second primary malignancy, (2) patients who missed follow-ups, and (3) patients who had non-complete clinical data (marital status, cause of death, survival month, tumor size, staging, and follow-up months). Patients were randomly distributed to a training or internal validation group and an external validation group. The study required no local ethical approvals or statements, as all data in the research were selected out of the SEER database.

### Variables and outcomes

Based on 14 clinical variables, we examined the age at diagnosis, race (Black, White and other, which including American Indian/Alaska Native and Asian/Pacific Islander), sex (female and male), marital status, years of diagnosis (2004–2009, 2009–2015, and 2016–2018), grade (I–III), histological subtype (papillary, follicular, medullary, or anaplastic), T stage (T1–T4), M stage (M0 or M1), N stage (N0–N1), tumor size, surgery, radiotherapy, and chemotherapy conditions. Patients who were widowed, divorced, separated, or bachelor (with a domestic partner or unmarried) were classified as unmarried. Regarding grading, grade I represented a highly differentiated cancer, grade II represented a moderately differentiated cancer, and grade III represented a poorly differentiated cancer. grade IV represented Undifferentiated cancer. Tumor diameters (0–10, 11–20, 21–40, and >40 mm) were translated into classification variables to test the linear hypothesis. There was no detailed information about radiotherapy regimens and chemotherapy drugs in the SEER database; therefore, these variables could not be further evaluated and controlled in this study. Finally, these TC’s variables (radiotherapy and chemotherapy) are used as dichotomous variables. The primary outcomes were OS and cancer-specific survival (CSS).The total survival time from TC diagnosis to TC-related or other causes of death as OS, whereas diagnosis to death or censoring as a result of TC was defined as CSS. For TNM staging, the 6-8th edition of the AJCC clinical staging guidelines was used for the study, which used the data from 2004-2018.

### Statistical analysis

The training cohort was used to construct the nomograms and develop the predictive model and risk stratification system. In contrast, the validation cohort was used to test the predictive model and risk stratification system. All eligible cases were randomized into the training and internal validation cohort (split 7:3) from 2004 to 2015. All eligible patients from 2016 to 2018 were used as the external validation group.

Cox Proportional Hazards Regression Models for each putative prognostic variable were used to calculate the associated 95% CIs and hazard ratios (HRs). Multivariate analysis included the relevant factors from a univariate analysis (P < 0.05). Besides, the statistical studies contributed to using the program SPSS 24 (SPSS, Chicago, IL). Based on the results of the multivariable analysis, the well-constructed nomogram may provide graphical risk predictions using the RMS and survival packages of R 4.0.2. The nomograms were validated both internally and externally. Nomograms were built as an intuitive scoring plot based on the traditional Cox proportional risk regression model. We combined the predictive power of conventional regression models with user-friendly and easy-to-use performance to construct a nomogram to predict patient survival. The TNM stage system and the net clinical advantages of the prediction model were further evaluated using a decision curve analysis (DCA).

Meanwhile, a risk categorization system was established based on each patient’s total nomogram score. The X-Tile program determined the best cut-off value for each patient’s total score (Robert L. Camp, Yale University, New Haven, Connecticut, USA). The patients were then divided into two prognostic categories based on the best cut-off value: the low-risk and high-risk groups. The Kaplan–Meier curves and log-rank test were also used to depict and compare the OS and CSS of patients with geriatric in various risk groups.

## Results

### General clinicopathological features

**Figure 1:**
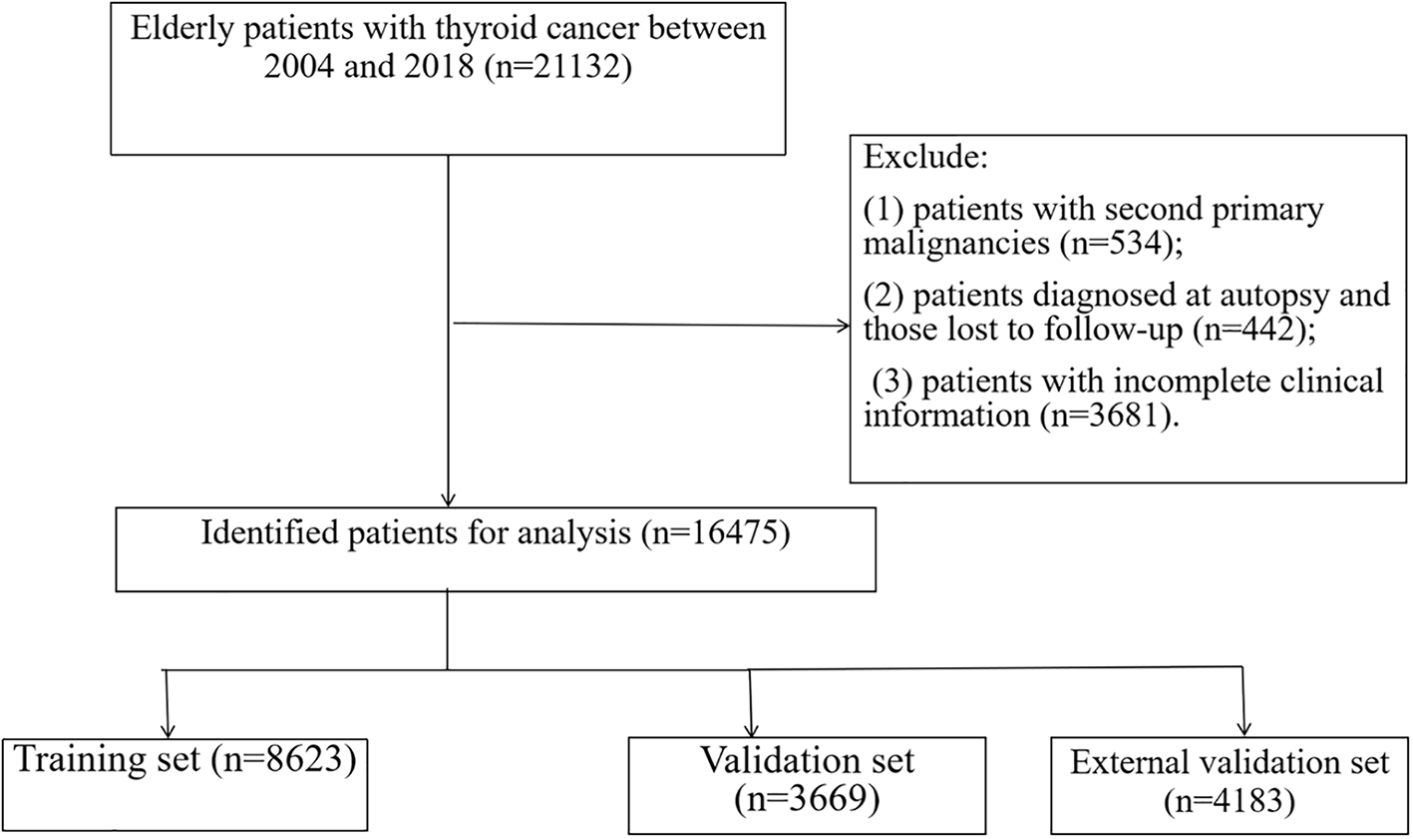
The flowchart of including and dividing patients.

Between 2004 and 2018, 16475 registered geriatric patients with TC were included from the SEER database according to the eligibility criteria. **Figure 1** illustrates the flowchart of the patient selection process. There were no significant differences in demographic information, tumor type, or treatment between the training and validation groups (**Table 1**), including the training group (n = 8623, diagnosed between 2004 and 2015), and the validation sample comprised 3,669 patients (n = 3669, diagnosed between 2004 and 2015). **Table S1** demonstrates the patients (n = 4183, diagnosed between 2016 and 2018) in the external validation group.

**Table 1:** Clinicopathological characteristics of elderly patients with thyroid cancer.

	ALL	Training cohort	Validation cohort	
	N = 12292	1 N = 8623	2 N = 3669	p
Age	72.6 (6.32)	72.6 (6.34)	72.6 (6.27)	0.714
Race				0.986
white	10259 (83.5%)	7195 (83.4%)	3064 (83.5%)	
black	690 (5.61%)	486 (5.64%)	204 (5.56%)	
other	1343 (10.9%)	942 (10.9%)	401 (10.9%)	
Sex				0.322
Male	4030 (32.8%)	2803 (32.5%)	1227 (33.4%)	
Female	8262 (67.2%)	5820 (67.5%)	2442 (66.6%)	
Marital				0.694
No	5127 (41.7%)	3607 (41.8%)	1520 (41.4%)	
Married	7165 (58.3%)	5016 (58.2%)	2149 (58.6%)	
Year of diagnosis				0.418
2004-2009	4799 (39.0%)	3346 (38.8%)	1453 (39.6%)	
2010-2015	7493 (61.0%)	5277 (61.2%)	2216 (60.4%)	
Histologic type				0.753
Papillary	10251 (83.4%)	7211 (83.6%)	3040 (82.9%)	
Follicular	1034 (8.41%)	717 (8.31%)	317 (8.64%)	
Medullary	626 (5.09%)	434 (5.03%)	192 (5.23%)	
Anaplastic	381 (3.10%)	261 (3.03%)	120 (3.27%)	
Grade				0.285
I	1721 (14.0%)	1195 (13.9%)	526 (14.3%)	
II	470 (3.82%)	311 (3.61%)	159 (4.33%)	
III	224 (1.82%)	158 (1.83%)	66 (1.80%)	
IV	479 (3.90%)	330 (3.83%)	149 (4.06%)	
Unknown	9398 (76.5%)	6629 (76.9%)	2769 (75.5%)	
T				0.197
T1	6524 (53.1%)	4628 (53.7%)	1896 (51.7%)	
T2	1695 (13.8%)	1172 (13.6%)	523 (14.3%)	
T3	2741 (22.3%)	1910 (22.2%)	831 (22.6%)	
T4	1332 (10.8%)	913 (10.6%)	419 (11.4%)	
N				0.142
N0	9684 (78.8%)	6796 (78.8%)	2888 (78.7%)	
N1a	1259 (10.2%)	905 (10.5%)	354 (9.65%)	
N1b	1349 (11.0%)	922 (10.7%)	427 (11.6%)	
M				1.000
M0	11723 (95.4%)	8224 (95.4%)	3499 (95.4%)	
M1	569 (4.63%)	399 (4.63%)	170 (4.63%)	
Tumor.size				0.360
0-10mm	4568 (37.2%)	3232 (37.5%)	1336 (36.4%)	
11-20mm	3114 (25.3%)	2201 (25.5%)	913 (24.9%)	
21-40mm	2828 (23.0%)	1958 (22.7%)	870 (23.7%)	
>40mm	1782 (14.5%)	1232 (14.3%)	550 (15.0%)	
Surgery				0.410
No	701 (5.70%)	474 (5.50%)	227 (6.19%)	
Lobectomy	1777 (14.5%)	1241 (14.4%)	536 (14.6%)	
Subtotal or near total thyroidectomy	428 (3.48%)	307 (3.56%)	121 (3.30%)	
Total thyroidectomy	9386 (76.4%)	6601 (76.6%)	2785 (75.9%)	
Chemotherapy				1.000
No/Unknown	12015 (97.7%)	8429 (97.8%)	3586 (97.7%)	
Yes	277 (2.25%)	194 (2.25%)	83 (2.26%)	
Radiation				0.490
No/Unknown	6871 (55.9%)	4838 (56.1%)	2033 (55.4%)	
Yes	5421 (44.1%)	3785 (43.9%)	1636 (44.6%)	
Survival months	76.5 (42.9)	76.6 (42.9)	76.0 (42.8)	0.474

In the whole study cohort, the average age was 72.6 (SD: 6.32) years; 8262 (67.2%) patients were female, and 4030 (32.8%) were male. Among all patients, the average age was 72.6 (SD: 6.34) in the training group and 72.6 (SD: 6.27) in the internal validation group. Most tumors (62.8%) were ≥1.0 cm in size. A total of 76.4% of patients received thyroidectomy, and 44.1% of patients received radioactive iodine. Additionally, 53.1% (6,524 out of 12,292), 13.8% (1,695 out of 12,292), 22.3% (2,741 out of 12,292), and 10.8% (1,332 out of 12,292) of patients had T1, T2, T3, and T4 tumors, respectively. Furthermore, 78.8% (9,684 of 12,292) of the patients were in the negative N stage, and 21.2% (4,129 of 12,292) were in the positive N stage. The average follow-up duration in the study cohort was 76.5 months (SD: 42.9). Moreover, 85.6% (83.8%–87.4%), 80.6% (79.1%–82.1%), and 78.7% (77.4%–80.1%) represented the 1-, 3-, and 5-year OS rates in the training group, respectively. The 1-, 3-, and 5-year OS rates in the validation group were 87.8% (85.4%–90.2%), 81.5% (79.3%–83.7%), and 78.8% (76.8%–80.8%), respectively.

### Univariate and multivariate analyses

In the training group, age, race, sex, marital status, years of diagnosis, histologic type, pathological grade, TNM stage, tumor size, surgery, radiation, and chemotherapy were all determined using the univariate Cox regression analysis with P < 0.05. Next, these characteristics were then examined in a multivariate Cox regression model (**Table 2**), which showed that the clinical features associated with survival included age (HR 1.076, 95% CI 1.07–1.082), sex (HR 0.685, 95% CI 0.63–0.745), marital status (HR 0.779, 95% CI 0.718–0.845), histologic type (papillary as a reference; medullary: HR 1.361, 95% CI 1.167–1.589; anaplastic: HR 1.671, 95% CI 1.243–2.245), tumor grade (grade I as a reference; grade III: HR 1.566, 95% CI 1.234–1.989; grade IV: HR 3.223, 95% CI 2.392–4.344), T stage (T1 as a reference; T4: HR 1.53, 95% CI 1.28–1.83), N stage (N0 as a reference; N1a: HR 1.174, 95% CI 1.032–1.335; N1b: HR 1.469, 95% CI 1.311–1.646), M stage (M0 as a reference; M1: HR 2.848, 95% CI 2.488–3.26), tumor size (0–10 mm as a reference; 21–40 mm: HR 1.481, 95% CI 1.143–1.669; >40 mm: HR1.639, 95% CI 1.369–1.963), and surgery procedure (no surgery as a reference; lobectomy. HR 0.441, 95% CI 0.378–0.516; subtotal or near total thyroidectomy: HR 0.524, 95% CI 0.42–0.653; total thyroidectomy: HR 0.403, 95% CI 0.354–0.459). These clinical prognostic variables were included in the constructed OS nomogram for further analysis. We also performed a competitive risk multiple analysis on patients who died from cancer (**Table 3**).

**Table 2:** Univariate and multivariate analyses of OS in training set.

	Univariate			Multivariate		
	HR	95%CI	P	HR	95%CI	P
Age	1.1	1.09-1.1	<0.001	1.076	1.07-1.082	<0.001
Sex						
Male						
Female	0.67	0.62-0.73	<0.001	0.685	0.63-0.745	<0.001
Race						
white						
black	1.18	1.01-1.37	0.04			
other	0.9	0.79-1.03	0.116			
Year of diagnosis						
2004-2009						
2010-2015	1	0.92-1.09	0.993			
Marital						
No						
Married	0.71	0.66-0.77	<0.001	0.779	0.718-0.845	<0.001
Histologic type						
Papillary						
Follicular	1.28	1.12-1.46	<0.001	0.957	0.83-1.102	0.54
Medullary	1.67	1.44-1.94	<0.001	1.361	1.167-1.589	<0.001
Anaplastic	18.5	16.1-21.26	<0.001	1.671	1.243-2.245	0.001
Grade						
I						
II	1.24	0.98-1.58	0.075	0.98	0.771-1.246	0.869
III	3.85	3.06-4.85	<0.001	1.566	1.234-1.989	<0.001
IV	15.69	13.3-18.51	<0.001	3.223	2.392-4.344	<0.001
Unknown	1.13	0.99-1.28	0.062	0.966	0.851-1.096	0.588
T						
T1						
T2	1.5	1.34-1.69	<0.001	0.914	0.742-1.126	0.397
T3	1.61	1.46-1.78	<0.001	0.961	0.821-1.126	0.622
T4	5.37	4.87-5.93	<0.001	1.53	1.28-1.83	<0.001
N						
N0						
N1a	1.38	1.22-1.56	<0.001	1.174	1.032-1.335	0.015
N1b	2.78	2.52-3.08	<0.001	1.469	1.311-1.646	<0.001
M						
M0						
M1	6.53	5.79-7.36	<0.001	2.848	2.488-3.26	<0.001
Tumor size						
0-10mm						
11-20mm	1.27	1.14-1.42	<0.001	1.107	0.987-1.243	0.083
21-40mm	1.93	1.74-2.14	<0.001	1.381	1.143-1.669	0.001
>40mm	3.77	3.39-4.19	<0.001	1.639	1.369-1.963	<0.001
Surgery						
No						
Lobectomy	0.18	0.16-0.21	<0.001	0.441	0.378-0.516	<0.001
Subtotal or near total thyroidectomy	0.21	0.17-0.26	<0.001	0.524	0.42-0.653	<0.001
Total thyroidectomy	0.17	0.15-0.19	<0.001	0.403	0.354-0.459	<0.001
Radiation						
No/Unknown						
Yes	1	0.92-1.07	0.911			
Chemotherapy						
No/Unknown						
Yes	7.5	6.38-8.81	<0.001			

**Table 3:** Multivariate Cox regression models predict cancer-specific mortality in elderly patients with thyroid cancer.

	CSM		
	HR	95%CI	P
Age	1.038	1.02 - 1.05	<0.001
Sex			
Male			
Female	0.990	0.83 - 1.18	0.91
Race			
white			
black	0.909	0.65 - 1.26	0.57
other	1.135	0.9 - 1.43	0.28
Year of diagnosis			
2004-2009			
2010-2015	0.806	0.68 - 0.96	0.013
Marital			
No			
Married	0.851	0.72 - 1.01	0.066
Histologic type			
Papillary			
Follicular	1.119	0.85 - 1.48	0.4
Medullary	2.320	1.8 - 2.98	<0.001
Anaplastic	1.381	0.94 - 2.03	0.099
Grade			
I			
II	1.347	0.83 - 2.17	0.22
III	2.762	1.87 - 4.07	<0.001
IV	5.185	3.3 - 8.14	<0.001
Unknown	1.068	0.79 - 1.45	0.67
T			
T1			
T2	1.166	0.75 - 1.81	0.5
T3	1.894	1.35 - 2.67	<0.001
T4	4.675	3.21 - 6.8	<0.001
N			
N0			
N1a	1.606	1.29 - 2	<0.001
N1b	1.447	1.16 - 1.8	<0.001
M			
M0	3.896	3.1 - 4.89	<0.001
M1			
Tumor size			
0-10mm			
11-20mm	1.392	1.01 - 1.92	0.046
21-40mm	1.910	1.28 - 2.84	<0.001
>40mm	2.461	1.68 - 3.61	<0.001
Surgery			
No			
Lobectomy	0.586	0.42 - 0.81	<0.001
Subtotal or near total thyroidectomy	0.816	0.54 - 1.23	0.33
Total thyroidectomy	0.561	0.43 - 0.74	<0.001
Radiation			
No/Unknown			
Yes	0.978	0.81 - 1.18	0.82
Chemotherapy			
No/Unknown			
Yes	1.210	0.9 - 1.62	0.2

### Nomogram development and validation

**Figure 2:**
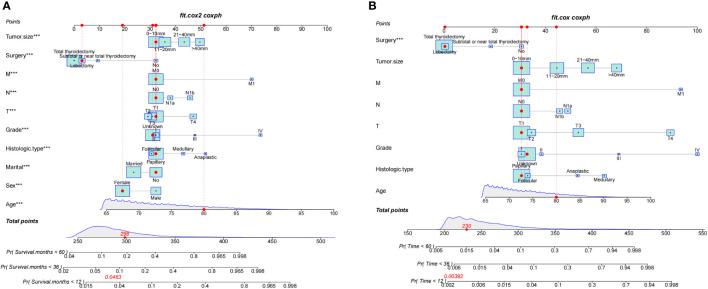
Nomograms for 1-, 3-, and 5-year OS **(A)** and CSS **(B)** of patients with TC. ***, a highly significant variable.

This study discovered 10 independent predictive factors based on the multivariate Cox regression results and generated a predictive OS nomogram. Age, sex, marital status, histologic type, tumor grade, T stage, N stage, M stage, tumor size, and operation are all shown in **Figure 2A**. Each clinical feature was assigned a score. The estimated 1-, 3-, and 5-year OS probabilities were easily calculated by adding the scores for all 10 clinical features and drawing a vertical line between the total score and the survival probability axis. Tumor grade and M stage were found to substantially impact prognosis, followed by surgical type, histologic type, T stage, tumor size, N stage, sex, race, and marital status on the nomogram. The training and validation groups had C-indices of 0.775 (95% CI: 0.785–0.765) and 0.776 (95% CI: 0.792–0.760), respectively.

**Figure 3:**
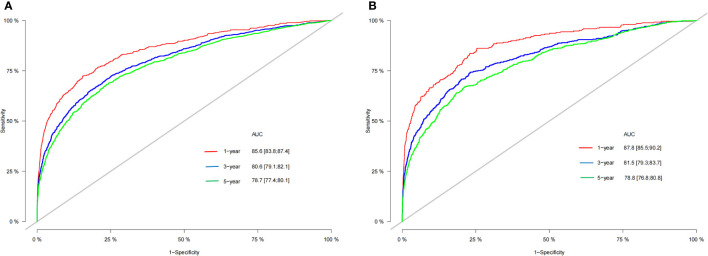
The AUC for OS of 1-, 3- and 5-year of training cohort **(A)** and validation cohort **(B)**.

**Figure 4:**
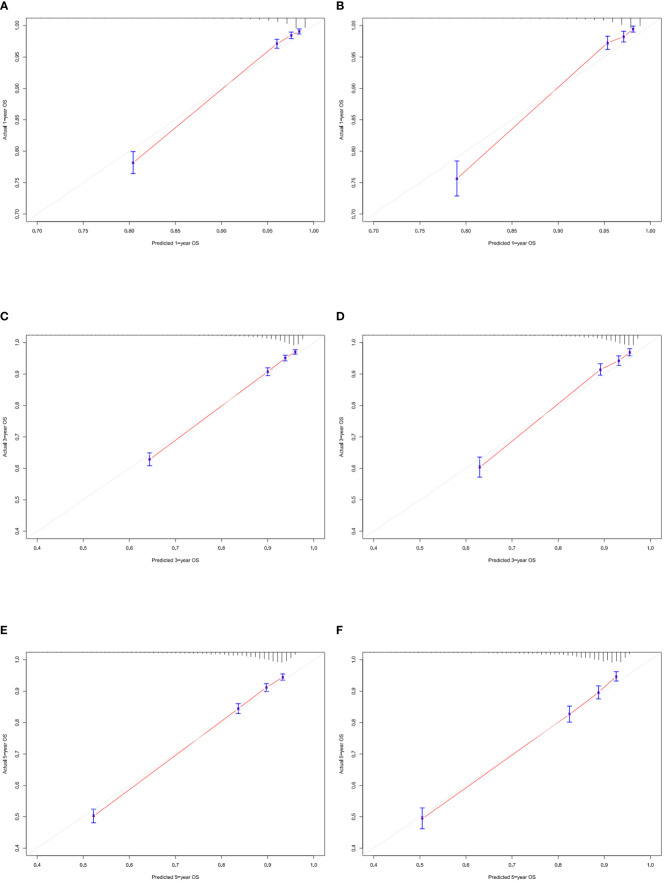
Calibration curves of nomogram. **(A–C)** For 1-, 3-, and 5-year OS in training cohort; **(D–F)** For 1-, 3-, and 5-year OS in validation cohort.

**Figure 5:**
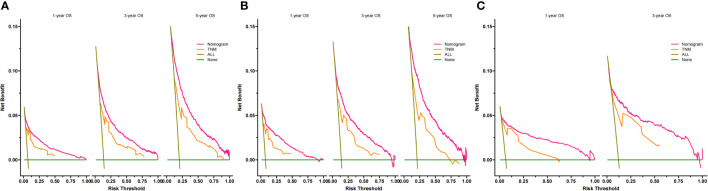
Decision curves of the nomogram predicting OS in training cohort **(A)**, validation cohort **(B)** and external validation cohort **(C)**. The y-axis represents the net benefit, and the x-axis represents the threshold probability. When the threshold probability is between 20% and 60%, the net benefit of the model exceeds all deaths or no deaths.

According to **Figure 3**, the training group’s 1-, 3-, and 5-year areas under the curve (AUCs) were 0.856, 0.806, and 0.787, respectively. On the other hand, the validation group’s 1-, 3-, and 5-year AUCs were, respectively, 0.878, 0.815, and 0.787. These findings demonstrated that the model prediction accuracy was high. Calibration curves of the training and validation groups used 1,000 bootstraps, suggesting high agreement between anticipated and actual outcomes (**Figure 4**). TNM staging was compared with the DCA curve of the training group to assess the clinical viability of the nomogram. According to the results, the nomogram was more vital in predicting 1-, 3-, and 5-year OS in patients with geriatric TC compared to TNM staging (**Figure 5**).

**Figure 6:**
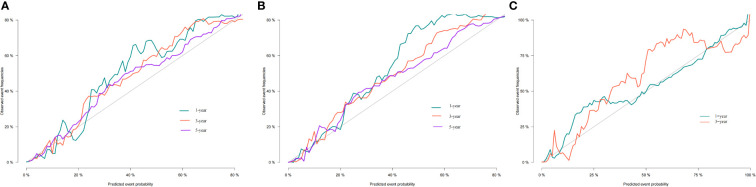
Calibration curves of nomogram. **(A)** For 1-, 3-, and 5-year CSS in training cohort; **(B)** For 1-, 3-, and 5-year CSS in validation cohort; **(C)** For 1-, 3-year CSS in external validation cohort.

Additionally, we constructed a competitive risk model to predict patients’ CSS (**Figure 2B**). The 1-, 3-, and 5-year C-indexes of the training group were 93.9, 92.1, and 90.5, respectively. The 1-, 3-, and 5-year C-indexes of the validation group were 95.5, 93.9, and 91.2, respectively. The 1- and 3-year C-indexes of the external validation group were 95.1 and 95.2, respectively. The calibration curve of the competitive risk model also showed that the predicted value is highly consistent with the actual observed value, suggesting that the model has good accuracy (**Figures 6A, B**). The calibration curve of external validation also showed that the model has good accuracy (**Figure 6C**).

### Risk stratification analysis

**Figure 7:**
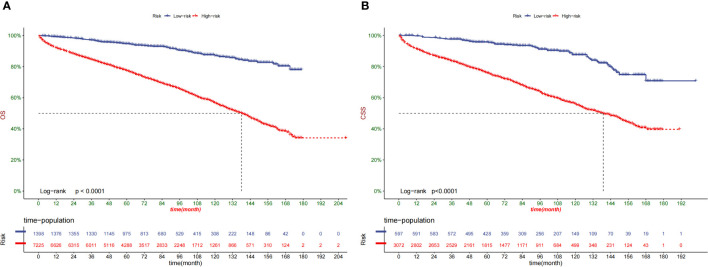
Kaplan–Meier curves of OS for patients in the low-, and high-risk groups in the training Cohort **(A)** and validation Cohort **(B)**.

Following the optimal cut-off value, patients were divided into two prognostic groups: the low-risk group (total score ≤ 24.9) and the high-risk group (total score > 24.9) (**Figures 7A, B**). According to the Kaplan–Meier curve, the risk stratification system could accurately recognize the training and validation cohorts from the OS. The high-risk patients had 1-, 3-, and 5-year OS rates of 91.8%, 84.1%, and 76.9%, respectively. On the other hand, the low-risk patients had 1-, 3-, and 5-year OS rates of 99.3%, 97.2%, and 79.50%, respectively.

### Effects of surgery on survival in different stratifications

**Figure 8:**
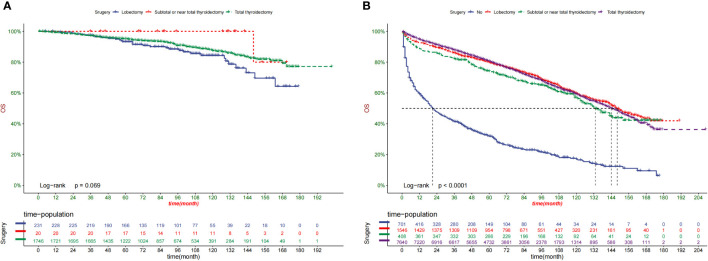
OS prediction of patients with different surgery in low- **(A)** and high-risk **(B)** group.

Kaplan–Meier curves were created for the low-risk and high-risk groups to further analyze the benefit of surgery in terms of survival (**Figures 8A, B**).In addition, the impact of different surgical methods on the survival probability of patients in the low-, and high-risk groups was summarized. In the low-risk group, almost everyone has undergone surgery (**Figure 8A**). A subset of patients in the high-risk group did not undergo surgery; that group had the lowest survival probability (**Figure 8B**).

### Construction of a web app for easy nomogram access

The online app may be found at https://zhangtingting.shinyapps.io/DynNomapp/ and is designed to help researchers and physicians determine patient survival probabilities.

## Discussion

In this study, 16475 individuals with geriatric TC were included. The histologic type, tumor grade, TNM stage, tumor size, and surgery were determined by the univariate and multivariate Cox analyses. The C-index and calibration charts were used to test the model and revealed good differentiation and calibration. According to DCA, our OS nomogram had a superior clinical net and a more excellent threshold probability range in predicting 1-, 3-, and 5-year OS in the training and validation groups than the usual TNM stage system. Meanwhile, the CSS nomogram was constructed using eight independent parameters: age, tumor size, grade, histologic type, surgery, T stage, N stage, and M stage. Although the prognosis of thyroid cancer varies considerably by histological type, inspired by previous studies (27), we included elderly patients with thyroid cancer of various tissue types and included tissue type as a variable in the regression analysis. Histologic type was included as an important variable in the prediction model’s construction with poorer survival for more aggressive histologic subtypes compared to papillary (**Table 2**, medullary: HR 1.361, 95%CI 1.167–1.589 and anaplastic: HR 1.671, 95% CI 1.243–2.245). In the online APP based on the model construction, clinicians can enter the patient’s tissue type. The survival prediction of patients with thyroid cancer including MTC is obtained.

Many scoring systems are used for predictive purposes. Despite a more simplified utilization in the clinic, only a stratified population risk assessment could be conducted for each patient (28). Nomograms are helpful tools for evaluating patient survival outcomes. Statistical modeling and risk quantification are used to handle the difficulty of balancing multiple factors. Their methodical methodology also eliminates the influence of individual physicians’ biases or aberrant clinical factors. Nomograms are more accurate than typical stage score methods (29–31). They may also be the most beneficial when the prospective advantages of additional therapy are unknown (32, 33). They are also great for personalized risk assessment and assisting clinicians with clinical care management when there are no definite guidelines.

To the best of our knowledge, this is the first study to describe the development and validation of a nomogram to forecast 5-year OS and CSS in TC-affected elderly individuals. Our nomograms exhibited good calibration and discrimination. The nomograms surpassed the accuracy of the TNM staging mechanism, as seen by the receiver operating characteristic curve. Our nomogram models are straightforward therapeutic aids that can support patient counseling and treatment individualization.

Our nomograms found several independent variables potentially impacting the outcome in elderly patients with TC. First, age is a significant factor for CSS in patients with TC (34). As a separate risk factor, patients with geriatric thyroid cancer have a reduced chance of surviving (35–37). Patients with geriatric TC have unique psychological features compared to younger patients including more comorbidities and shorter life expectancy. Less life expectancy, more comorbidities, and a shorter life expectancy are among these factors. The previous version of the AJCC staging system divided people by 45 years, but the 8th version utilizes 55 years for the same purpose. Age is recognized as a significant prognostic factor independent of the cut-off number.

The gender disparity in TC prevalence has also been thoroughly documented (38). Women are more likely to develop TC than men, although men have worse clinical outcomes (39). The findings of our patients with geriatric were similar to those in prior investigations. In addition to the above-mentioned characteristics, marital status, histologic type, tumor grade, T stage, N stage, M stage, tumor size, and surgery were significant prognostic indicators. However, we discovered that neither gender nor marital status was a risk factor for the 1-, 3-, or 5-year CSS.

The association of marital status and survival was explored in many tumors, including breast cancer, rectal cancer, and non-small cell lung cancer (40–42).We identified that marital status was an independent prognostic factor in the univariate analysis, with married patients having a decreased chance of mortality (43). After adjusting for demographic and clinical characteristics, married patients were shown to have a lower mortality risk than unmarried patients. In a prior study concentrating on differentiated patients with TC, Shi et al. have discovered that single individuals had a higher risk of tumor death (44). In a study of breast cancer patients aged ≥70 years, marriage has been found to provide higher protection from poorer prognosis (45). According to a study that analyzed more than a million patients diagnosed with various diseases, unmarried individuals have had a greater chance of metastatic cancer and death from cancer (46). Consistent with the findings of the above researches, our results found that marriage was a factor associated with superior survival. There are two possibilities that could explain why married patients live longer than unmarried patients. On the one hand, these married patients were overseen by their spouses for frequent physical checkups before being diagnosed, which helps detect TC early. Meanwhile spouses may also provide more economic support for subsequent treatments. On the other hand, cancer patients are more than four times more likely to suffer from psychological disorders (47). After being diagnosed with cancer, married persons had reduced despair and psychological suffering, which may be attributed to the encouragement and support from their spouses (43, 48).

Our prediction model may be used in clinical practice to estimate patient survival by alerting doctors about the predicted advantages of various therapies. In this study, we found that for elderly patients with TC, almost all patients in the low-risk group undergo thyroidectomy. Regardless of surgical method, patients undergoing surgery have better overall survival. Most high-risk patients did not undergo surgery.

Patients with TC have a variety of risk variables, including age, grade, TNM stage, and tumor size. Elderly patients are more likely to have comorbidities and therefore surgery could reduce the OS rate of patients. However, for the first time, our study found that surgery is advantageous to cancer-specific higher-risk senior individuals with TC (P < 0.0001) but not to low-risk groups (P = 0.069), providing doctors with suggestions for extending their patients’ lives.

This study had some limitations. First, the nomograms were created using historical data. As a result, there was a possibility of selection bias. Second, the SEER does not cover all factors; hence, only 14 variables were included in our analysis. Some critical factors were not included, such as the degree of surgery, radioiodine dose, thyrotropin suppression, etc. Third, as a retrospective cohort research, selection bias might have existed because only patients with comprehensive information on essential characteristics were included. Fourth, the majority of the participants in this study were Americans. As a result, prospective clinical pilot studies are needed to see if the findings can be generalized to different groups.

## Conclusion

Based on this study, the first applicable nomograms were created, along with an online application that predicts the personalized long-term OS and CSS of geriatric TC patients. The nomogram performed effectively and had great accuracy and dependability. It is the first nomogram based on a large number of patients with external validation.

## Data availability statement

The original contributions presented in the study are included in the article/**Supplementary Material**. Further inquiries can be directed to the corresponding authors.

## Author contributions

T-TZ, YY, and JZ all helped with the idea and design. The data were analyzed by T-TZ, YY, and Y-JK. The manuscript was written by T-TZ. A critical revision of the manuscript was contributed by XW and YF. The final version of the work has been reviewed and approved by all authors.
