# Supplementary material for: A visualized dynamic prediction model for survival of patients with geriatric thyroid cancer: A population-based study
Source: Front Endocrinol (Lausanne). 2022 Dec 9;13:1038041. doi: 10.3389/fendo.2022.1038041 (PMC9780441; doi:10.3389/fendo.2022.1038041)
Supplement: Supplementary file 1 [file Table_1.docx]

**Table S1. Clinicopathological characteristics of elderly patients with thyroid cancer in** **external validation cohort.**

|  | **ALL**  **N=4183** |
| --- | --- |
| Age | 72.1 (6.05) |
| Race |  |
| white | 3434 (82.1%) |
| black | 241 (5.76%) |
| other | 508 (12.1%) |
| Sex |  |
| Male | 1489 (35.6%) |
| Female | 2694 (64.4%) |
| Marital |  |
| No | 1693 (40.5%) |
| Married | 2490 (59.5%) |
| Histologic type |  |
| Papillary | 3546 (84.8%) |
| Follicular | 271 (6.48%) |
| Medullary | 204 (4.88%) |
| Anaplastic | 162 (3.87%) |
| Grade |  |
| I | 311 (7.43%) |
| II | 65 (1.55%) |
| III | 62 (1.48%) |
| IV | 154 (3.68%) |
| Unknown | 3591 (85.8%) |
| T |  |
| T1 | 2207 (52.8%) |
| T2 | 668 (16.0%) |
| T3 | 900 (21.5%) |
| T4 | 408 (9.75%) |
| N |  |
| N0 | 3043 (72.7%) |
| N1a | 529 (12.6%) |
| N1b | 611 (14.6%) |
| M |  |
| M0 | 3964 (94.8%) |
| M1 | 219 (5.24%) |
| Tumor size |  |
| 0-10mm | 1324 (31.7%) |
| 11-20mm | 1191 (28.5%) |
| 21-40mm | 1038 (24.8%) |
| >40mm | 630 (15.1%) |
| Surgery |  |
| No | 349 (8.34%) |
| Lobectomy | 669 (16.0%) |
| Subtotal or near total thyroidectomy | 73 (1.75%) |
| Total thyroidectomy | 3092 (73.9%) |
| Chemotherapy |  |
| No/Unknown | 4050 (96.8%) |
| Yes | 133 (3.18%) |
| Radiation |  |
| No/Unknown | 2627 (62.8%) |
| Yes | 1556 (37.2%) |
